# Successful transitioning is a matter of the Heart: Integrated Care for Grown-Up Congenital Heart Disease

**Published:** 2012-09-04

**Authors:** K. Viktoria Stein

**Affiliations:** Institute of Social Medicine, Center for Public Health, Medical University of Vienna, Austria

**Keywords:** transition from paediatric to adult services, GUCH, implementation of guidelines, integrated care centres

## Abstract

**Purpose:**

This study offers a comprehensive overview over the existing guidelines for GUCH/ACHD care and synthesises the recommendations made over the past decade, developing them into an integrated care concept for GUCH/ACHD patients. Its aim is to emphasise the need for more coordinated action of paediatric and adult specialists, professional and patients organisations to lobby for a concerted implementation of the guidelines for GUCH/ACHD management and an organised transitioning process.

**Context:**

More than a decade ago, discussions picked up on the adequate management of a challenging new patient group: persons with ‘Grown-Up Congenital Heart Disease’ (GUCH), also known as ‘Adult Congenital Heart Disease’ (ACHD) in North America. The various authors acknowledged the demand for highly specialised and trained professionals who could provide the wide array of services needed for this patient group, with a systematic and multi-disciplinary approach. First experiences have already been gathered throughout the 1990s in Canada and the UK. Since then, the technological and medical advances in paediatric cardiology, cardiac surgery and related medical fields have improved the health outcomes even further, to the extent that 85%–95% of children with congenital heart disease (CHD) survive into adulthood. However, the efforts to implement the necessary managerial, transitioning and vocational training requirements have not been afforded equal focus.

**Data sources:**

A literature review of the existing guidelines for the management of GUCH patients from national and international cardiology associations, expert interviews.

**Case description:**

The key problem in the management of GUCH patients is a lack of understanding the importance of a coordinated transitioning process from paediatric to adult care services. Neither the paediatric nor the adult specialists have the proper training for the care of these patients, the former lacking experience with adult patients the latter not knowing the complex indication of congenital heart disease. In the different guidelines (e.g. from the American Heart Association or the European Society of Cardiology), it is acknowledged that cooperation and communication between specialists and settings and a managed transitioning process are paramount. In this case, the focus is laid on developing an integrated care model based on the existing medical guidelines and the requirements a transition process demands. Adolescents with CHD and their parents need to be prepared to adapt to the demands of an adult life. They need information on working and educational options, family planning, and what complications may be expected. Also, the adolescents need to learn to take over the responsibility for their own life and health—independent of their parents. These are just the most pressing of the challenges GUCH patients face.

**(Preliminary) conclusions:**

Even though specialised GUCH/ACHD centres exist in many countries, they are too few in numbers to effectively and adequately service the whole population and provide high quality training. A lack of coordination and communication between paediatric and adult health care service providers results in patients being lost in transition from paediatric to adult care settings. This counteracts the excellent services children with congenital heart disease receive nowadays, and which have lead to the need of specialised adult service in the first place. It is a waste of time and resources if the efforts made in the paediatric care setting are not followed up adequately once the patients are grown up. This is a classic setting for implementing integrated care and this study offers a model, based on the available medical guidelines to do so.

